# Relationship between albumin-corrected anion gap and non-alcoholic fatty liver disease: a cross-sectional analysis of NHANES 2017–2018

**DOI:** 10.3389/fmed.2025.1518540

**Published:** 2025-03-12

**Authors:** Ning Bai, Ting Ying, Dejian Li, Aiguo Liu

**Affiliations:** ^1^Department of Gastroenterology, Huaihe Hospital of Henan University, Kaifeng, China; ^2^Research Institute of Digital and Intelligent Orthopedics, Shanghai Pudong Hospital, Fudan University Pudong Medical Center, Shanghai, China; ^3^Department of Orthopedics, The First Affiliated Hospital of Henan University, Kaifeng, China

**Keywords:** non-alcoholic fatty liver disease, albumin-corrected anion gap, age-related differences, cross-sectional study, NHANES

## Abstract

**Objectives:**

The objective of this study was to examine the correlation between the albumin-corrected anion gap (ACAG) and non-alcoholic fatty liver disease (NAFLD) using data from the National Health and Nutrition Examination Survey (NHANES) 2017–2018.

**Methods:**

A cross-sectional analysis was conducted, comprising 4,379 participants, who were stratified into two groups: those with NAFLD and those without NAFLD. The baseline characteristics were compared using the most appropriate statistical tests. The relationship between ACAG levels and NAFLD was assessed using generalized linear models, with adjustments made for potential confounding factors. The analysis of threshold effects was conducted using piecewise regression. Furthermore, the relationship between ACAG and NAFLD was investigated in different age groups.

**Results:**

The mean age of participants with non-alcoholic fatty liver disease (NAFLD) was significantly higher than that of non-NAFLD participants (48.88 vs. 43.46 years, *p* < 0.001). The presence of NAFLD was associated with higher levels of ACAG (18.80 ± 0.24 vs. 18.10 ± 0.19, *p* < 0.001). In fully adjusted models, each 1-unit increase in ACAG was associated with a significantly increased risk of NAFLD in participants under 60 years old (*β*: 0.87, 95% CI: 0.05, 1.69, *p* < 0.05). In younger participants, elevated NAFLD risk was observed in those with higher ACAG quartiles (*P* for trend <0.05). In contrast, no significant associations were identified in participants aged 60 years and older (*P* for trend >0.05), suggesting the presence of age-specific differences in the relationship between ACAG and NAFLD.

**Conclusion:**

The impact of ACAG on NAFLD is significantly correlated, especially in the age group, where elevated levels of ACAG are associated with increased risk of NAFLD in young people. ACAG may be a potential and reliable biomarker for predicting NAFLD risk in clinical assessment, but its implementation should consider the patient’s age factor.

## Introduction

Non-alcoholic fatty liver disease (NAFLD) is a prevalent chronic liver condition, defined by the excessive accumulation of fat within the liver. It affects approximately 25% of the global adult population (1, 2). This condition encompasses a spectrum of liver disorders, ranging from simple steatosis to non-alcoholic steatohepatitis (NASH), which is characterized by inflammation, hepatocyte ballooning, and varying degrees of fibrosis (3). The progression from NAFLD to NASH markedly elevates the risk of developing advanced liver diseases, including cirrhosis and hepatocellular carcinoma (4). This poses a significant public health challenge (5, 6). NAFLD is closely associated with several metabolic disorders, including obesity, type 2 diabetes mellitus (T2DM), and cardiovascular diseases. These conditions share common pathophysiological mechanisms, including insulin resistance and systemic inflammation (7–9). Moreover, NAFLD is acknowledged as an independent risk factor for cardiovascular disease and chronic kidney disease, contributing to elevated morbidity and mortality rates in affected individuals (10, 11).

The progression of NAFLD is frequently associated with metabolic dysregulation, particularly the emergence of insulin resistance, which gives rise to lipotoxicity, mitochondrial dysfunction, and oxidative stress, thereby intensifying inflammation and fibrosis (12–15). Recent studies have indicated that alterations in the acid–base balance may also exert an influence on the progression of NAFLD and its associated complications, including liver fibrosis and cirrhosis. An acidic microenvironment has been demonstrated to augment inflammatory responses and facilitate fibrogenesis in hepatic tissues (16). Therefore, it is of great significance to identify an acid-related biomarker and to monitor its levels in order to prevent the escalation of NAFLD in a timely manner.

The anion gap (AG) is a crucial parameter that reflects the body’s acid–base balance. Primarily, it is used to assess the type of metabolic acidosis and its potential causes. An elevated AG value is typically indicative of the accumulation of lactic acid, ketone bodies, or other organic acids, which frequently occurs in conjunction with various metabolic disorders (17). In recent years, there has been a notable increase in research activity concerning the relationship between AG and NAFLD. It has been demonstrated that AG values are frequently significantly abnormal in patients with NAFLD, which may be closely associated with metabolic abnormalities and oxidative stress within the body (18, 19). Moreover, since albumin is a pivotal element in the calculation of AG, the clinical interpretation of AG may be hindered in patients with hypoalbuminemia (20). Accordingly, the albumin-corrected anion gap (ACAG) has been put forth as a means of more accurately assessing the acid–base balance in patients with liver diseases. The research on the correlation between ACAG and NAFLD indicates that higher ACAG levels may be closely associated with the severity and progression of NAFLD, particularly in patients with concurrent hypoalbuminemia, where ACAG more accurately reflects the abnormalities in their acid–base balance (21). These findings indicate that ACAG plays an important role in the evaluation of acid–base disorders and may also serve as a potential marker for assessing disease progression and the risk of complications in NAFLD patients.

Therefore, a cross-sectional study was conducted based on the National Health and Nutrition Examination Survey (NHANES) (2017–2018) with the objective of determining the correlation between ACAG and NAFLD. Furthermore, we sought to ascertain whether age influenced the correlation between ACAG and NAFLD. The objective was to explore the relationship between ACAG and NAFLD and to investigate whether ACAG levels have potential value in predicting the risk of NAFLD or fibrosis.

## Methods

### Study design and population

The research data utilized in this study were obtained from the National Health and Nutrition Examination Survey (NHANES), covering the years 2017 to 2018. This survey is a project managed by the Centers for Disease Control and Prevention (CDC) in the United States,^1^ aiming to evaluate the health and nutritional conditions of the American populace, with assessments conducted biennially. The protocol for NHANES has received approval from the Ethics Review Committee at the National Health Statistics Research Center, ensuring that all participants have given their written informed consent. Detailed information regarding the ethical aspects and the consent process can be found on the NHANES website.^2^

**Figure 1:**
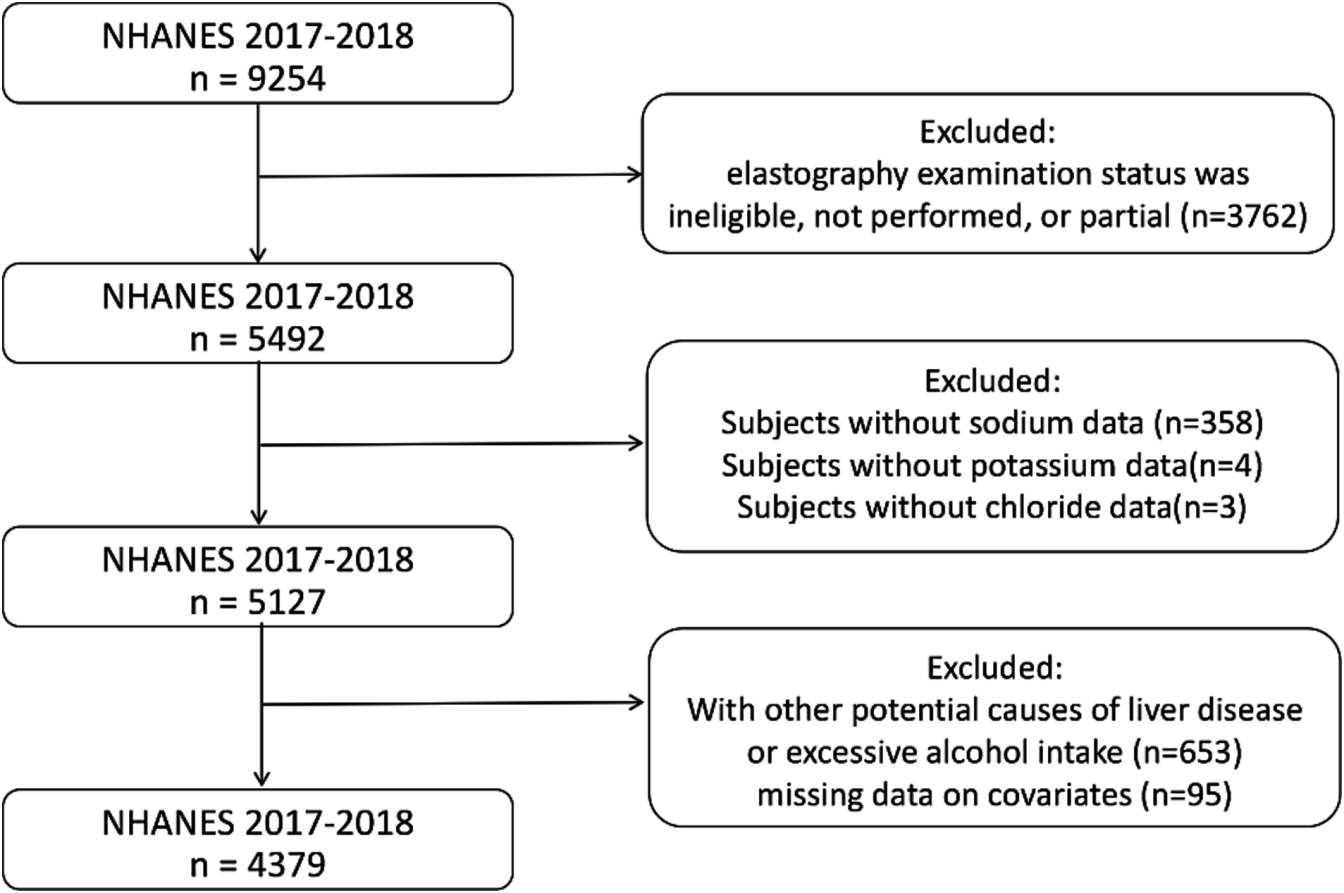
Flowchart of participants selection. NHANES, National Health and Nutrition Examination Survey.

A total of 9,254 samples were included in this cycle. The exclusion criteria included the following: an ineligible, not performed, or partial elastography examination status (*n* = 3,762); missing sodium data (*n* = 358); missing potassium data (*n* = 4); missing chloride data (*n* = 3); serologic positivity for viral hepatitis (*n* = 124); excessive alcohol intake, defined as more than four or five standard drinks per day (*n* = 529); and missing data on covariates (*n* = 95). Subsequently, 4,379 samples were included in the analysis (Figure 1).

### Definition of NAFLD and significant liver fibrosis (SLF)

The liver ultrasound transient elastography has become a widely adopted non-invasive method for assessing liver health, having received FDA approval. This study employed the FibroScan® model 502 V2 Touch (Echosens, Paris, France), equipped with medium or extra-large probes, to perform elastography examinations in NHANES Mobile Examination Centres with the objective of evaluating liver stiffness and steatosis. It was required that participants fast for a minimum of three hours, provide at least ten valid measurements, and have an interquartile range-to-median ratio of liver stiffness measurements (LSM) less than 30% for the examinations to be considered complete. A diagnosis of NAFLD was made when a controlled attenuation parameter (CAP) value of 263 dB/m or above was observed, while SLF was indicated by a median liver stiffness of 8.2 kPa or above (22–24).

### Variable extraction and ACAG calculation

The demographic characteristics of the participants included age, sex, race, educational attainment, body mass index (BMI) and poverty income ratio (PIR). Information regarding smoking status was obtained from self-report questionnaires in the MECs. This included whether the participant was a former smoker (i.e., had smoked at least 100 cigarettes in their lifetime and still smoked cigarettes at the time of the survey) or a never smoker (i.e., had not smoked at least 100 cigarettes in their lifetime). Furthermore, data regarding diabetes were also collected via self-report questionnaires. Serum biochemistry profiles were obtained for the following parameters: total calcium, alanine aminotransferase (ALT), aspartate aminotransferase (AST), high-density lipoprotein (HDL) cholesterol, vitamin C, sodium, potassium, chloride, bicarbonate, and albumin. To avoid the potential for collinearity, sodium, potassium, chloride, bicarbonate, and albumin were employed exclusively for the calculation of ACAG and were subsequently excluded from further statistical analyses.

The AG was calculated in accordance with the following equation: AG (mmol/L) is calculated according to the following equation: AG (mmol/L) = (sodium + potassium) - (chloride + bicarbonate). The ACAG was calculated according to the following formula: ACAG (mmol/L) is calculated according to the following formula: ACAG (mmol/L) = [4.4 - albumin (g/dl)] * 2.5 + AG (25).

### Statistical analysis

The baseline characteristics of participants were summarized using weighted analyses to account for the complex sampling design of NHANES. Continuous variables were expressed as weighted means ± standard error (SE) and categorical variables as weighted frequencies (percentages). Group comparisons for continuous variables were performed using weighted linear regression models for normally distributed data and weighted quantile regression for non-normally distributed data. Differences in categorical variables were evaluated with weighted chi-square tests. To explore the linear relationship between ACAG and NAFLD, we employed multivariate linear regression models, with subgroup analyses stratified by sex to identify variations across different populations. Non-linear relationships were investigated using smooth curve fittings and generalized additive models; if an inflection point was detected, it was calculated using two-piecewise linear regression models with a recursive algorithm. Association analyses utilized generalized linear models (GLMs) and logistic regression, while threshold effects were assessed through piecewise regression. Trend analyses were conducted to calculate *P*-trend values using quartile-based models, and subgroup analyses were adjusted for covariates in multivariable models. Statistical significance was defined as *p*-values less than 0.05, and all analyses were performed using R software (version 4.0.3^3^) and EmpowerStats (version 6.0^4^).

## Results

### Participant characteristics

Of the 4,379 participants, 2,409 were classified as non-NAFLD, while 1,970 exhibited NAFLD. Of these, 1,700 were found to have no SLF, while 270 exhibited SLF (Table 1). A statistically significant difference in age was observed between the groups, with participants diagnosed with NAFLD being older (mean age: 48.88 years) compared to those without the condition (mean age: 39.20 years; *p* < 0.001). The gender distribution revealed no significant difference between the groups, although a higher percentage of males were present in the NAFLD group with SLF in comparison to their non-NAFLD counterparts (53.84% vs. 41.76%). There was a significant discrepancy in educational attainment, with a greater proportion of participants in the non-NAFLD group having attained a high school or higher level of education (84.01% vs. 73.58%, *p* < 0.001).

**Table 1:** Baseline characteristics of participants in NHANES 2017–2018.

Variable	Overall (*n* = 4,379)	Non-NAFLD (*n* = 2,409)		NAFLD (*n* = 1970)			
			*p* value^b^	Total	Without SLF (*n* = 1700)	With SLF (*n* = 270)	*p* value^c^
Age (year)	43.46 ± 0.65	39.20 ± 0.66	<0.001	48.88 ± 0.80	48.57 ± 0.71	51.03 ± 1.93	0.141
Sex, *n* (%)*^a^*			<0.001				0.738
Male	2011 (46.26%)	1,018 (41.76%)		993 (51.99%)	850 (51.72%)	143 (53.84%)	
Female	2,368 (53.74%)	1,391 (58.24%)		977 (48.01%)	850 (48.28%)	127 (46.16%)	
Education level, *n* (%)*^a^*			<0.001				0.715
Under high school	1,500 (21.84%)	971 (26.42%)		529 (15.99%)	462 (16.12%)	67 (15.12%)	
High school or above high school	2,879 (78.16%)	1,438 (73.58%)		1,441 (84.01%)	1,238 (83.88%)	203 (84.88%)	
PIR, *n* (%)*^a^*			0.999				0.167
< 1	1,237 (21.16%)	679 (21.16%)		558 (21.15%)	486 (20.60%)	72 (24.98%)	
≥ 1	3,142 (78.84%)	1730 (78.84%)		1,412 (78.85%)	1,214 (79.40%)	198 (75.02%)	
Race/Ethnicity, *n* (%)*^a^*			<0.001				0.907
Non-hispanic white	660 (10.15%)	288 (7.92%)		372 (13.00%)	316 (12.92%)	56 (13.55%)	
Non-hispanic black	400 (7.05%)	220 (7.54%)		180 (6.41%)	155 (6.22%)	25 (7.73%)	
Mexican American	1,457 (61.28%)	797 (61.71%)		660 (60.73%)	570 (61.00%)	90 (58.91%)	
Other Hispanic	947 (10.67%)	580 (11.94%)		367 (9.04%)	319 (9.15%)	48 (8.31%)	
Other races	915 (10.85%)	524 (10.89%)		391 (10.82%)	340 (10.71%)	51 (11.50%)	
BMI, *n* (%)*^a^*			<0.001				<0.001
< 25 kg/m^2^	1,472 (32.66%)	1,273 (52.15%)		199 (7.82%)	187 (8.59%)	12 (2.63%)	
25 ≤ BMI < 30 kg/m^2^	1,328 (29.81%)	704 (29.91%)		624 (29.68%)	580 (32.09%)	44 (13.42%)	
≥ 30 kg/m^2^	1,579 (37.53%)	432 (17.94%)		1,147 (62.50%)	933 (59.32%)	214 (83.95%)	
Smoke status, *n* (%)*^a^*			<0.001				0.361
Never	3,095 (68.00%)	1810 (71.97%)		1,285 (62.94%)	1,121 (63.31%)	164 (60.43%)	
Current	511 (11.70%)	273 (10.99%)		238 (12.60%)	209 (12.76%)	29 (11.49%)	
Former	773 (20.30%)	326 (17.04%)		447 (24.46%)	370 (23.93%)	77 (28.08%)	
Diabetes, *n* (%)*^a^*			<0.001				<0.001
Yes	529 (9.39%)	147 (3.55%)		382 (16.83%)	287 (14.31%)	95 (33.84%)	
No	3,850 (90.61%)	2,262 (96.45%)		1,588 (83.17%)	1,413 (85.69%)	175 (66.16%)	
AST (U/L)	21.40 ± 0.20	20.36 ± 0.34	0.002	22.72 ± 0.40	21.86 ± 0.40	28.51 ± 1.64	0.001
ALT (U/L)	21.76 ± 0.29	18.08 ± 0.32	<0.001	26.46 ± 0.72	25.34 ± 0.79	33.97 ± 1.80	<0.001
HDL cholesterol (mmol/L)	1.39 ± 0.01	1.48 ± 0.01	<0.001	1.27 ± 0.01	1.28 ± 0.01	1.20 ± 0.04	0.051
ACAG (mmol/L)	18.41 ± 0.21	18.10 ± 0.19	<0.001	18.80 ± 0.24	18.69 ± 0.23	19.57 ± 0.38	0.005
Serum vitamin C (umol/L)	52.58 ± 1.37	56.29 ± 1.37	<0.001	47.84 ± 1.92	48.90 ± 26.16	43.04 ± 25.55	0.002
Serum total calcium (mmol/L)	2.33 ± 0.02	2.34 ± 0.01	0.063	2.33 ± 0.01	2.33 ± 0.01	2.33 ± 0.01	0.832

Values are means ± SE for continuous variables. *p* Value was calculated by weighted linear regression model for continuous variables and weighted chi-square test was performed for categorical variables. ^a^Unweighted frequency counts and weighted percentages are shown. ^b^NAFLD vs. Non-NAFLD. ^c^NAFLD without SLF vs. with SLF. NAFLD non-alcoholic fatty liver disease, SLF significant liver fibrosis, ACAG albumin-corrected anion gap, PIR poverty income ratio, BMI body mass index, HDL cholesterol high-density lipoprotein cholesterol, AST aspartate aminotransferase, ALT alanine aminotransferase.

The prevalence of diabetes was notably higher in the NAFLD group (16.83% vs. 3.55%, *p* < 0.001). The BMI classifications revealed significant disparities between the two groups. A substantial proportion of the non-NAFLD participants had a BMI of less than 25 kg/m^2^ (52.15%), while the majority of the NAFLD participants had a BMI of 30 kg/m^2^ or greater (62.50%), with a statistically significant difference between the two groups (*p* < 0.001). The prevalence of current smokers was similar across the groups. However, former smokers were more prevalent in the NAFLD group (24.46% vs. 17.04%, *p* < 0.001).

Biochemical parameters demonstrated notable discrepancies. Participants with NAFLD exhibited elevated levels of liver enzymes, with AST and ALT being higher in the NAFLD group (AST: 22.72 U/L; ALT: 26.46 U/L) compared to non-NAFLD participants (AST: 20.36 U/L; ALT: 18.08 U/L; both *p* < 0.05). Furthermore, participants with NAFLD exhibited lower HDL cholesterol levels (1.27 mmol/L) compared to those without NAFLD (1.48 mmol/L, *p* < 0.001). Additionally, serum vitamin C levels were significantly lower in the NAFLD group (47.84 umol/L vs. 56.29 umol/L, *p* < 0.001).

### Association between ACAG and NAFLD

As demonstrated in Table 2, in Model 1, which remains unadjusted, each 1 unit increase in ACAG is associated with a *β* coefficient of 1.13 (95% CI: 0.46, 1.79), indicating a significant positive relationship with NAFLD (*p* < 0.01). Model 2, adjusted for age, sex, education level, PIR, and race/ethnicity, shows a similar trend with a *β* value of 1.20 (95% CI: 0.53, 1.87) (*p* < 0.01). However, in Model 3, which further includes adjustments for smoking status, diabetes, BMI, HDL cholesterol, AST, ALT, SLF, serum total calcium, and serum vitamin C, the association strengthens but becomes less pronounced, yielding a *β* coefficient of 0.72 (95% CI: 0.07, 1.36) (*p* < 0.05).

**Table 2:** Association between ACAG and NAFLD.

ACAG	β (95% CI)		
	Model 1	Model 2	Model 3
Per 1 increment	1.13 (0.46, 1.79)**	1.20 (0.53, 1.87)**	0.72 (0.07, 1.36) *
Quartile			
Q1 (<17.05)	Reference	Reference	Reference
Q2 (17.05 to<18.45)	5.64 (0.71, 10.58)*	6.15 (1.23, 11.08)*	5.03 (0.40, 9.66)*
Q3 (18.45 to<19.90)	10.11 (5.19, 15.03)***	10.45 (5.52, 15.39) ***	6.87 (2.21, 11.53)**
Q4 (≥19.90)	9.33 (4.57, 14.09)***	10.32 (5.52, 15.12)***	7.04 (2.46, 11.62)**
*P* for trend	<0.0001	<0.0001	0.0036

Model 1 was adjusted for none. Model 2 was adjusted for Age, Sex, Education level, PIR, Race/Ethnicity. Model 3 was adjusted for Age, Sex, Education level, PIR, Race/Ethnicity, Smoke status, Diabetes, BMI, HDL cholesterol, AST, ALT, Serum total calcium, Serum vitamin C, SLF. β: CAP. *p < 0.05, **p < 0.01, ***p < 0.001. NAFLD non-alcoholic fatty liver disease, ACAG albumin-corrected anion gap, PIR poverty income ratio, BMI body mass index, HDL cholesterol high-density lipoprotein cholesterol, AST aspartate aminotransferase, ALT alanine aminotransferase, SLF significant liver fibrosis, CAP controlled attenuation parameter.

Regarding quartile analysis, participants in the second quartile (ACAG 17.05 to <18.45) exhibit a β of 5.64 (95% CI: 0.71, 10.58) in Model 1, indicating increased NAFLD risk, while Model 2 enhances this estimate to 6.15 (95% CI: 1.23, 11.08) (*p* < 0.05). In quartile 3 (ACAG 18.45 to <19.90), the association reaches 10.11 (95% CI: 5.19, 15.03) in Model 1 and 10.45 (95% CI: 5.52, 15.39) in Model 2, both statistically significant (*p* < 0.001). The highest quartile (≥19.90) reflects *β* coefficients of 9.33 (95% CI: 4.57, 14.09) and 10.32 (95% CI: 5.52, 15.12) in Models 1 and 2, respectively (*p* < 0.001). *P* for trend analyses across all models yield significant results (*p* < 0.0001 for Models 1 and 2, and *p* = 0.0036 for Model 3), indicating a robust relationship between ACAG levels and the prevalence of NAFLD.

### Identification of nonlinear relationship between ACAG and NAFLD

**Figure 2:**
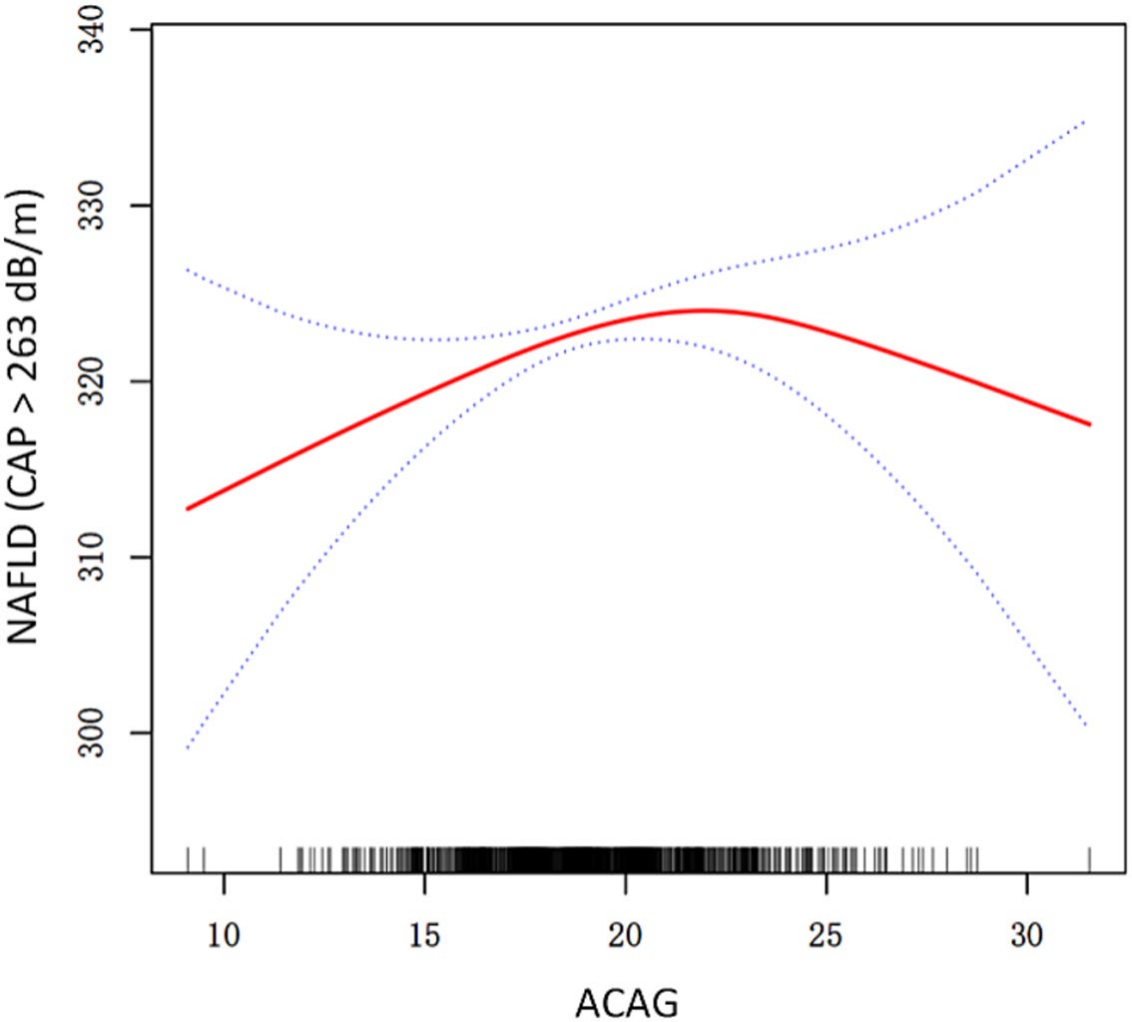
A threshold, nonlinear association between ACAG and NAFLD. Solid rad line represents the smooth curve fit between variables. Dashed line represent the 95% of confidence interval from the fit. All adjusted for Age, Sex, Education level, PIR, Race/Ethnicity, Smoke status, Diabetes, BMI, HDL cholesterol, AST, ALT, Serum total calcium, Serum vitamin C. NAFLD non-alcoholic fatty liver disease, ACAG albumin-corrected anion gap, PIR poverty income ratio, BMI body mass index, HDL cholesterol high-density lipoprotein cholesterol, AST aspartate aminotransferase, ALT alanine aminotransferase, CAP controlled attenuation parameter.

The threshold effect of ACAG on NAFLD, revealing a significant non-linear relationship that suggests an inverted U-shaped association (Table 3; Figure 2). In Model I, the linear analysis indicates a *β* coefficient of 0.39 (95% CI: −0.25, 1.02) with a *p*-value of 0.2349, suggesting no significant linear correlation between ACAG and the prevalence of NAFLD. However, upon further analysis in Model II, a turning point (K) of 23.05 is identified. For ACAG values below this threshold (ACAG < K), there is a positive association with NAFLD, reflected in a β of 0.86 (95% CI: 0.11, 1.60) and a statistically significant *p*-value of 0.0238. Conversely, for ACAG values at or above this turning point (ACAG ≥ K), the association shifts to negative, demonstrating a *β* of −3.16 (95% CI: −6.12, −0.21) with *p* = 0.0360. The logarithm likelihood ratio test (LRT) shows a *p*-value of 0.015, indicating that Model II significantly differs from Model I, strengthening the evidence for a non-linear relationship. The 95% confidence interval for the turning point ranges from 21.30 to 23.30, further substantiating the identified ACAG threshold.

**Table 3:** Threshold effect analysis of ACAG and NAFLD.

Model	β (95% CI)	*P* value
Model I		
One line effect	0.39 (−0.25, 1.02)	0.2349
Model II		
Turning point (K)	23.05	
ACAG<K	0.86 (0.11, 1.60)	0.0238
ACAG> = K	−3.16 (−6.12, −0.21)	0.0360
*P* value for LRT test*	0.015	
95% Cl for Turning point	21.30, 23.30	

Data were presented as β (95% CI) CAP *p* value; Model I, linear analysis; Model II, non-linear analysis. Adjusted for Age, Sex, Education level, PIR, Race/Ethnicity, Smoke status, Diabetes, BMI, HDL cholesterol, AST, ALT, Serum total calcium, Serum vitamin C. Restricted cubic spline smoothing were applied. *p < 0.05 indicates that Model II is significantly different from Model I. NAFLD non-alcoholic fatty liver disease, ACAG albumin-corrected anion gap, PIR poverty income ratio, BMI body mass index, HDL cholesterol high-density lipoprotein cholesterol, AST aspartate aminotransferase, ALT alanine aminotransferase, CAP controlled attenuation parameter, Cl confidence interval, LRT logarithm likelihood ratio test.

### Association between ACAG and NAFLD across different age groups

In individuals aged less than 60 years, there is a robust positive correlation between ACAG and NAFLD in all models (Table 4). Specifically, for each 1 increment in ACAG, the *β* coefficients are 1.86 (95% CI: 0.98, 2.75) in Model 1, 1.95 (95% CI: 1.06, 2.84) in Model 2, and 0.87 (95% CI: 0.05, 1.69) in Model 3, with the first two models achieving significance at *p* < 0.001. Quartile analyses in this age group show significant increases in *β* values across higher quartiles of ACAG, particularly notable in Q3 (*β* = 11.55, 95% CI: 5.37, 17.73; *p* < 0.001) and Q4 (β = 12.75, 95% CI: 6.67, 18.83; *p* < 0.001), with a clear trend (*p* < 0.001). In contrast, individuals aged 60 years and older demonstrate minimal associations between ACAG and NAFLD, with the β coefficients for each increment being non-significant across all models (0.08 to 0.19), indicating a lack of association. For the quartile analysis, the β values remain close to zero, with Q2, Q3, and Q4 demonstrating no significant increase in risk, and the *p*-values for trend do not reach significance (*p* = 0.2959 to 0.8515).

**Table 4:** Association between ACAG and NAFLD in different models among age groups.

ACAG	β (95% CI)		
	Model 1	Model 2	Model 3
Aged <60 years			
Per 1 increment	1.86 (0.98, 2.75)***	1.95 (1.06, 2.84)***	0.87 (0.05, 1.69)*
Q1 (<16.80)	Reference	Reference	Reference
Q2 (16.80 to<18.20)	7.32 (1.11, 13.53)*	7.90 (1.70, 14.10)*	5.56 (−0.01, 11.13)
Q3 (18.20 to<19.55)	11.55 (5.37, 17.73)***	11.93 (5.75, 18.10)***	7.02 (1.44, 12.59)*
Q4 (≥19.55)	12.75 (6.67, 18.83)***	14.00 (7.88, 20.12)***	6.86 (1.25, 12.46)*
*P* for trend	<0.0001	<0.0001	0.0223
Aged ≥60 years			
Per 1 increment	0.08 (−0.93, 1.10)	0.19 (−0.83, 1.21)	−0.46 (−1.44, 0.53)
Q1 (<16.80)	Reference	Reference	Reference
Q2 (16.80 to<18.20)	2.41 (−5.73, 10.55)	2.72 (−5.43, 10.86)	−1.20 (−8.93, 6.53)
Q3 (18.20 to<19.55)	7.32 (−0.81, 15.45)	7.36 (−0.91, 15.62)	1.11 (−6.75, 8.97)
Q4 (≥19.55)	3.53 (−4.14, 11.21)	4.31 (−3.51, 12.14)	−1.32 (−8.85, 6.22)
*P* for trend	0.2959	0.2361	0.8515

Model 1 was adjusted for none. Model 2 was adjusted for Age, Sex, Education level, PIR, Race/Ethnicity. Model 3 was adjusted for Age, Sex, Education level, PIR, Race/Ethnicity, Smoke status, Diabetes, BMI, HDL cholesterol, AST, ALT, Serum total calcium, Serum vitamin C, SLF. β: CAP. *p < 0.05, **p < 0.01, ***p < 0.001. NAFLD non-alcoholic fatty liver disease, ACAG albumin-corrected anion gap, PIR poverty income ratio, BMI body mass index, HDL cholesterol high-density lipoprotein cholesterol, AST aspartate aminotransferase, ALT alanine aminotransferase, SLF significant liver fibrosis, CAP controlled attenuation parameter.

## Discussion

The present study revealed the intricate relationship between ACAG and NAFLD through a comprehensive analysis of the results obtained from a variety of models. The results presented in Table 1 indicate that an increase in ACAG is significantly associated with an elevated risk of NAFLD, particularly in younger individuals, where this association is particularly pronounced. Table 2 provides further evidence of the effectiveness of ACAG as a potential biomarker, after adjustment for multiple confounding factors. Table 3 clearly demonstrates an inverse U-shaped relationship between ACAG and NAFLD risk. ACAG below a certain threshold is associated with a significant increase in risk, while above this threshold, there is a decrease in risk. This indicates that ACAG plays a dual role in NAFLD assessment. Finally, the age stratification analysis in Table 4 indicates that the effect of ACAG on NAFLD is significant in the population under 60 years of age, while no correlation is observed in the population aged 60 and above. These findings indicate that ACAG is not only an important indicator for assessing NAFLD risk, but also emphasize the moderating role of age in this relationship.

Our study shares similarities with Lu et al.’s research on the association between ACAG and NAFLD, while also demonstrating significant differences (21). Both studies confirm that ACAG is an independent risk factor for NAFLD and employ various statistical methods to assess the significance of this relationship. The published study emphasizes the role of waist circumference in moderating the relationship between ACAG and NAFLD across different populations, while our research explores the non-linear relationship between ACAG levels and NAFLD risk across different age groups, revealing an inverse U-shaped trend. These differences indicate that while both studies focus on the association between ACAG and NAFLD, our research further expands this field by providing a more comprehensive perspective on the effects of ACAG across diverse populations, offering new insights for clinical practice.

The association between ACAG and NAFLD involves multiple potential mechanisms that can be explained through metabolic disorders and inflammatory responses. Firstly, an elevation in ACAG typically reflects an imbalance in the acid–base status of the body, particularly in the context of metabolic syndrome and insulin resistance. In such scenarios, the levels of lactate and ketone bodies tend to increase, which may exert direct toxic effects on the liver, thereby promoting lipid deposition and leading to the development of NAFLD (26). Furthermore, increased ACAG levels may also correlate with adipocyte dysfunction. Research indicates that metabolic abnormalities can lead to enhanced fat synthesis coupled with reduced fat oxidation, resulting in excessive fat accumulation in the liver and further promoting NAFLD progression (27). Secondly, the elevation of ACAG is closely associated with increased oxidative stress, which is recognized as a significant mechanism in the progression of NAFLD. Oxidative stress arises from the overproduction of free radicals, leading to lipid peroxidation and subsequent damage to liver cells, thereby triggering inflammatory responses (28). This chronic inflammatory state not only exacerbates hepatic steatosis but may also contribute to liver fibrosis and further hepatic injury (29, 30). The role of pro-inflammatory cytokines, such as TNF-*α* and IL-6, is particularly noteworthy, as they are extensively involved in the pathophysiology of NAFLD. Studies have shown that these cytokines are significantly elevated in NAFLD patients, and their presence correlates with the severity of liver inflammation and damage (31).

The observed inverted U-shaped relationship between ACAG and NAFLD risk may reflect dual biological mechanisms. At lower ACAG levels (<23.05 mmol/L), elevated AG likely signifies subclinical metabolic acidosis, promoting lipotoxicity, oxidative stress, and hepatic lipid accumulation. However, at higher ACAG levels (≥23.05 mmol/L), hypoalbuminemia (common in advanced liver disease) may dominate, reducing AG correction accuracy and masking acidosis effects. Additionally, compensatory mechanisms (e.g., renal bicarbonate retention) in chronic acidosis could mitigate hepatic damage, explaining the attenuated risk at higher ACAG (15, 25, 28).

This study examined the association between ACAG and significant fibrosis in NAFLD using a large sample study with nationally representative data and adjusted for potential confounders to improve the reliability of the results. However, this study has some limitations. Firstly, this is a cross-sectional study and cannot determine the causal relationship between ACAG and NAFLD. Second, including only individuals with complete data and excluding those with missing data may introduce bias for other potential confounders, affecting the accurate assessment of the true association. Third, the participants are based on the general population in the United States and may not be generalizable to other countries due to potential differences in genetics, language, culture and environmental factors between countries and regions. While weighted analyses were applied to baseline characteristics to reflect national estimates, subsequent multivariable models utilized unweighted data to prioritize biological inference. Future studies with larger samples should validate these associations using weighted approaches to ensure generalizability. Finally, there is currently no consensus regarding the critical value for the detection of steatosis and the evaluation of liver fibrosis using LSM. Consequently, the limitations of our research may be constrained by the identification of NAFLD with or without significant fibrosis.

In conclusion, the impact of ACAG on NAFLD is significantly correlated, especially in the age group, where elevated levels of ACAG are associated with increased risk of NAFLD in young people. ACAG may be a potential and reliable biomarker for predicting NAFLD risk in clinical assessment, but its implementation should consider the patient’s age factor.

## Data Availability

The original contributions presented in the study are included in the article/supplementary material, further inquiries can be directed to the corresponding authors.

## Footnotes

^1^ https://www.cdc.gov/nchs/nhanes/?CDC_AAref_Val=https://www.cdc.gov/nchs/nhanes/index.htm

^2^ https://wwwn.cdc.gov/nchs/nhanes/ContinuousNhanes/Documents.aspx?BeginYear=2017

^3^ https://www.R-project.org

^4^ https://www.empowerstats.net/analysis/install/index.php
